# The impact of using different ancestral reference populations in assessing crossbred population admixture and influence on performance

**DOI:** 10.3389/fgene.2022.910998

**Published:** 2022-09-26

**Authors:** Mohd A. Jaafar, Bradley J. Heins, Chad Dechow, Heather J. Huson

**Affiliations:** ^1^ Department of Animal Science, Cornell University, Ithaca, NY, United States; ^2^ West Central Research and Outreach Centre, University of Minnesota, Morris, MN, United States; ^3^ Department of Animal Science, Penn State University, State College, University Park, PA, United States

**Keywords:** crossbreed, dairy cattle, admixture, breed ancestry, ProCROSS, grazecross, viking red

## Abstract

Crossbreeding is a process in which animals from different breeds are mated together. The animals produced will exhibit a combination of both additive and non-additive genetic improvement from parental breeds that increase heterozygosity and negate inbreeding depression. However, crossbreeding may also break up the unique and often beneficial gene combinations in parental breeds, possibly reducing performance potential as the benefits of heterosis depends on the type of crossbreeding systems used and heritability of the traits. This effect of crossbreeding, especially on the genome architecture, is still poorly understood with respect to 3-breed crossbreeding systems. Thus, this study examined variation in genomic ancestry estimations relative to pedigree-based estimations and correlated breed composition to key production and health traits. Two rotational crossbred populations, referenced as ProCROSS and Grazecross were assessed and totaled 607 crossbred cattle. ProCROSS is a product of rotational crossbreeding of Viking Red (VKR), Holstein (HOL), and Montbeliarde (MON). In contrast, Grazecross consists of Viking Red (VKR), Normande (NOR), and Jersey (JER). Both breeding programs were aimed at capitalizing on the positive effect of heterosis. The VKR is a marketing term for Swedish Red, Danish Red, and Finnish Ayrshire breed which complicated breed determination. Therefore, genomic breed composition estimates were compared using two different representations of VKR, one of which was based on parents used in the crossing system and a second based on genotypes from the ancestral breeds that comprise VKR. Variation of breed composition estimates were assessed between pedigree and genome-based predictions. Lastly, Genomic estimations were correlated with production and health traits by comparing extreme performance groups to identify the relationship between breed ancestry and performance. With the exception of the JER breed composition in Grazecross, all other estimates of the purebred contribution to the ProCROSS and Grazecross showed a significant difference in their genomic breed estimation when using the VKR ancestral versus the VKR parental reference populations for admixture analysis. These observations were expected given the different relationship of each VKR representation to the crossbred cattle. Further analysis showed that regardless of which VKR reference population was used, the degree of MON and HOL breed composition plays a significant role in milk and fat production in ProCROSS, while the degree of VKR and NOR ancestry were related to improved health performance in Grazecross. In all, identifying the most appropriate and informative animals to use as reference animals in admixture analysis is an important factor when interpreting results of relationship and population structure, but some degree of uncertainty exists when assessing the relationship of breed composition to phenotypic performance.

## 1 Introduction

Within domesticated animals, “pure” breeds have been developed and are a recognized population of individuals displaying specific attributes. A purebred animal is bred from parents of the same breed and is expected to inherit characteristics attributed to the breed that are likely under extreme selective pressure or possibly fixed within the breed, ensuring the propagation of these breed-specific traits in future generations. In contrast, crossbred animals, or animals bred from parents of differing breeds, will exhibit a combination of characteristics from both parental breeds. Intentional breeding of purebred or crossbred animals has inherent challenges and advantages with respect to offspring expressing the desired traits of mated individuals and managing inbreeding depression. To this end, much research has been invested in studying the development of pure breeds and crossbreds.

Globally, dairy cattle have undergone natural and artificial selection with varying degrees of selective pressure resulting in many well recognized pure breeds of cattle and less characterized, but often environmentally adapted, indigenous or admixed cattle populations (Vanraden and Sanders, 2003; Stella et al., 2010; Zhao et al., 2015; Gautason et al., 2021). While the intense selection of purebred cattle has created breeds exceptionally known for milk production like the Holstein breed, milk solids in the Jersey, or superior health traits in the Norwegian Red breed, it has also led to inbreeding depression (Pryce et al., 2014; Gurgul et al., 2016; Dechow and Hansen, 2017: Gautason et al., 2021). Crossbreeding is commonly seen as a method of producing high production animals well-adapted to local environments and a tool to mitigate inbreeding depression (Mbole-Kariuki et al., 2014; Leroy et al., 2016).

In particular, the Holstein breed plays an important role in the dairy industry as a pure breed, noted for its exceptional production and its contribution to many crossbreeding programs similarly aiming to increase production (Vanraden and Sanders, 2003). Despite the breed’s notoriety for superior production, it was also noted for its declining reproductive performance up until approximately 2010 (VanRaden, 2017; Berry, 2018). Prior to 2010, fertility was inadvertently selected against due to its negative correlation to production traits under extreme selection, with poor fertility likely exacerbated by inbreeding depression (Berry et al., 2014). Inbreeding depression is the consequence of accumulating deleterious mutations inherited from common ancestors in the lineage and is often expressed when in a homozygous state. As intensive selection propagates homozygosity across the genome to stabilize trait selection within the breed, crossbreeding increases genomic diversity with resulting heterosis or hybrid vigor in offspring. Extensive experimentation and research have been and continue to be conducted in the dairy industry to explore the benefits and drawbacks of crossbreeding, including optimization of breeds to cross and the identification of breed influence on performance (Touchberry, 1992; McAllister et al., 1994; Heins et al., 2006a).

Multiple crossbreeding schemes have been explored with differing use of breeds as well as the number of breeds used in a program and how many generations crossbreeding continoues. Another common consideration is whether to continue breeding among the crossbred animals generated or to breed future generations to purebred animals. One such crossbreeding system found to monopolize on heterosis is a 3-breed rotational crossbreeding system using complementary breeds (Dechow and Hansen, 2017). The 3-breed crossbreeding rotation of complementary breeds was found to maintain the highest level of heterosis (86%) due to the relationship of bulls and cows mated being more remote. One such example of a 3-breed rotational cross known as ProCROSS, was developed and is marketed by ProCROSS International, owned by parent companies, VikingGenetics and Coopex Montbeliarde (Randers, Denmark and Roulans, France). This system starts by crossbreeding a pure Holstein (HOL) cow to a Viking Red (VKR) bull. The first generation (F1) progeny heifers are then mated to pure Montbeliarde (MON) bulls. Each successive generation of crossbred progeny is subsequently bred to bulls from HOL, VKR, and MON in a rotational pattern. The HOL, VKR, and MON were selected due to their historical trait improvements, with the HOL breed contributing milk volume and solid content to the 3-breed rotation and the MON and VKR breeds contributing strength, health, and fertility performance. Extensive research investigating the rotation of the 3-breeds and performance comparison to pure Holstein has been conducted (Heins et al., 2006a, Heins et al., 2006b, Heins et al., 2008a, Heins et al., 2008b, Heins et al., 2010, Heins et al., 2011, Heins et al., 2012, Heins, 2019). In addition, another crossbred population, known as Grazecross, was designed for low-input grazing systems. Grazecross consists of a 3-breed rotation of Jersey (JER), crossed to Normande (NOR), crossed to VKR. The JER is well known for its milk solid content but more diminutive stature than HOL, while NOR breeds are reported to produce high milk protein content with outstanding grazing ability (Heins et al., 2012).

Of particular note in the ProCROSS and Grazecross systems is the use of VKR cattle as developed and marketed by Viking Genetics (Randers, Denmark). The VKR cattle developed by Viking Genetics in the 1980s combined the genetic improvement programs of the previously separate Swedish Red and White, Finnish Ayrshire, and Danish Red populations. The idea of including the VKR in the crossbreeding program may increase the level of heterosis in ProCROSS and Grazecross by introducing new variant that are not routinely used in the United States. However, the effect of this mixed breed on crossbred genome architecture is poorly understood. Often the unique haplotypes or gene combinations created within a purebred population are signature to the purebred breed themselves and give rise to the crossbred performance. However, crossbreeding may also break up the unique and often beneficial gene combinations in purebred breeds, possibly reducing performance potential as the benefits of heterosis depend on the type of crossbreeding systems used and traits heritability.

This study focused on understanding the impact of using different reference populations in determining ancestry in admixed populations and how this influences global genome ancestry estimates. ProCROSS and Grazecross cattle and respective ancestral reference populations were the focus of our study and provided a unique opportunity to explore the impact of reference populations by assessing two different datasets to represent VKR ancestry, one being a combination of Swedish Red (SWD), Danish Red (DNR), and Finnish Ayrshire (FAY), and the other being commercially marketed VKR bulls used as sires in the breeding program. The first objective of this study was to characterize the genomic ancestry and assess variation in results dependent upon the reference population used. We expected the commercial VKR population to give a more exact representation of breed composition for the ProCROSS and Grazecross than the VKR ancestral reference panel due to the close relationship between commercial VKR parental animals and the crossbred populations. This study’s second aim was to compare breed estimation variation between pedigree records and genomic breed estimations using the two different VKR reference populations. Lastly, we explored the effect of global breed estimation and the influence of ancestry on production traits of milk volume, protein, fat, and the health trait of somatic cell score in the ProCROSS and Grazecross. The effect of reference population on the correlation between breed composition and performance was similarly assessed. Our results demonstrate how the use of specific animals representing the reference populations in an admixture study can alter results of breed composition in admixed animals. Therefore methology, including reference populations used and how this may affect interpretation, should be reviewed in research publications before considering applications to crossbreeding programs.

## 2 Materials and methods

### 2.1 Datasets

The ProCROSS and Grazecross data were from the University of Minnesota West Central Research and Outreach Center (UMN) dairy herd in Morris, Minnesota, United States. Data included 378 ProCROSS and 229 Grazecross cattle genotypes, three major production traits (Milk, Fat, and Protein Yield), and one health trait (Somatic Cell Score). Other relevant information such as sire and dam information, birth date, and recent sire breed were also included in these datasets. Both groups of admixed cattle have birthdates spanning from 2003 to 2018.

As described in the introduction, both cattle herds are produced through rotational crossbreeding, where ProCROSS cattle are generated through the rotational mating of cattle representing VKR, HOL, and MON breeds to hybrid cows. In contrast, Grazecross cattle utilize VKR, NOR, and JER. A more detailed description of the crossbreeding rotations are in Pereira and Heins (2019). To investigate the admixture of ProCROSS and Grazecross, cattle representing the purebred and admixed breeds used in the two rotational systems were needed for comparison. Table 1 summarizes the datasets used in this study with their respective numbers and source. The SNP data of all animals studied were obtained through bovine genotyping array kits, BovineHD DNA Analysis Kit (HD150K), BovineSNP50 DNA Analysis BeadChip (50K) and CLARIFIDE^®^ 50k (ZL5) (Illumina- Neogen, Lansing, MI, United States Clarifide–Zoetis, San Diego, CA, United States). In this study, two different datasets were used to represent the VKR population which has its own admixed origin as well. The first VKR representation included the 13 bulls marketed as VKR which were directly used in the breeding program for the ProCROSS and Grazecross in the study. These bulls were predominantly SWD and DNR crosses. The genotypes of the 13 bulls were provided by Viking Genetics (Randers, Denmark). The second VKR representation was the VKR’s ancestral breeds consisting of four DNR, 23 SWD and 27 FAY cattle, totaling 54 animals. While the DNR was from parental individuals, the SWD and FAY genotypes were purebred populations from Sweden and Finland, respectively (Iso-Touru et al., 2016; Upadhyay et al., 2019). The HOL genotypes were similarly directly related to the two admixed populations and represented animals used for breeding in the admixed populations or purebred counterparts within the same herds. The JER, MON, and NOR genotypes were obtained from Gautier et al. (2010) and represented purebred animals from the United States (JER) and Europe (MON and NOR), respectively.

**TABLE 1:** Summary of cattle samples by population, respective breeds, number of animals and genotype source.

Population	Breed	Sample (n)^a^	SNP^b^ chip	Origin of the data
Admixed	ProCROSS	378	HD150K/50K	UMN**^c^**
	Grazecross	229	HD150K/50K	UMN**^c^**
Ancestral	Holstein	92	HD150K/50K/ZL5^d^	UMN**^c^**
	Jersey	73	HD150K/50K	Gautier et al., 2010, Illumina^®^
	Montbéliarde	34	HD150K/50K	Gautier et al., 2010, Illumina^®^
	Normande	35	HD150K/50K	Gautier et al., 2010, Illumina^®^
	Swedish red	23	HD150K	Upadhyay et al. (2019)
	Finnish ayrshire	27	50K	Iso-Touru et al. (2016)
	Danish red	4	50K	Viking genetics
	Viking red	13	50K	Viking genetics

a Samples call rate >90%.

b SNP, Single Nucleotide Polymorphism.

c UMN, University of Minnesota.

d Zoetis low-density chip, version 5.

### 2.2 Filtering and quality control of genomic data

The total number of SNP markers available in the merged datasets for ProCROSS and Grazecross admixed populations with their ancestral populations were 156,731 and 149,030, respectively. However, the number of SNPs genotyped per animal varied considerably due to the differing densities of the genotyping platforms and potential laboratory batch effects in genotyping. In the ProCROSS dataset, 36% (*n* = 137) were genotyped with Bovine150K, while the rest (*n* = 241) were genotyped with BovineSNP50 platform. Grazecross animals were genotyped using the BovineSNP50 DNA Analysis BeadChip (50K) (*n* = 59) or BovineHD DNA Analysis Kit (HD150K) (*n* = 170). The SNP used for analysis were identified based on those common across genotyping platforms and passing quality control thresholds. Given the crossbred nature of much of the population and limited genotypes available for many of the purebred samples, imputation was not pursued. Quality control (QC) analyses for both sets of autosomal SNPs were calculated using SNP and Variation Suite (SVS) v8.x (Golden Helix, Inc., Bozeman, MT). Various thresholds for quality control measures were examined in an effort to maximize the number of SNP available for analysis while narrowing the SNPs used to ones informative for differentiating admixed and ancestry populations. This involved the evaluation of Minor Allele Frequencies (MAF) between 0.05 and 0.10 and SNP call rate between 0.6 and 0.95. The effects of each different threshold combination were assessed through Principal Component Analysis (PCA) diagrams. The selected thresholds (MAF: >0.05, SNPs call rate: >95%) resulted in tighter clustering of individuals within a population and relatively clear separation between ancestral and admixed populations (Supplementary Figure S1). In addition, the SNPs left were further pruned for linkage disequilibrium (LD) using a threshold of *r* ^*2*^ > 0.75. The SNPs were also excluded if they were unmapped to the UMD 3.1 bovine genome assembly (Zimin et al., 2009) or mapped to sex chromosomes.

### 2.3 Principal component analysis and estimation of fixation index

Genomic data were analyzed through PCA in SVS (Golden Helix, Inc., Bozeman, MT) with an additive model identifying the first 10 principal components. Besides identifying the SNP quality thresholds to maximize the number of SNPs used in the analysis, PCA was also used to assess population structure within admixed populations and compare to ancestral breeds. Analyses were conducted to confirm the relationship of the ancestral populations to the admixed population and identify if any substructure existed within the crossbred population. In total, six different datasets were analyzed by PCA, including two PCA for ProCROSS, two PCA for Grazecross, one PCA comparing VKR to their ancestral breeds, and one using all individuals representing VKR (parental and ancestral) with admixed populations and other purebreds. Within the two PCA each for ProCROSS and Grazecross, the reference population used to represent VKR cattle was alternated. One PCA for each admixed population used founding breeds of Viking Red, including SWD, FAY, and DNR, whereas the other PCA for each admixed population used cattle marketed as VKR, which were sires of animals within the admixed populations. The fifth PCA compared the marketed VKR with their ancestral breeds as noted above, to characterize the sub-structure related to VKR cattle. The other PCA incorporated all animals representing VKR, including the parental sires marketed as VKR and the individuals from the ancestral breeds. This PCA was run to identify any substructure related to VKR or how VKR clustered compared to the other purebred breeds or admixed population when combined. Estimation of fixation index (F_ST_) was based on Wright’s F statistic using SVS v8.8.5 (Golden Helix, Inc., Bozeman, MT) to investigate the genetic difference between the populations.

### 2.4 Genomic breed composition estimation

The genomic-based breed composition was estimated from genomic data using a maximum likelihood model implemented in ADMIXTURE 1.23 software (Alexander et al., 2009). PLINK software version 1.9 (Chang et al., 2015) was used to generate data input files for ADMIXTURE. This analysis identified genomic breed composition in the admixed populations that was used to compare with pedigree estimations of breed composition. The same datasets used in PCA analysis were analyzed using unsupervised clustering analysis with different K values where K represented the expected number of genetic clusters or ancestral populations. Due to nontypical breeds being detected in the pedigree information, such as JER in ProCROSS, an additional admixture analysis was run with all animals from the PCA datasets, including both admixture populations and all potential ancestral breed populations using *K* = 5 and 7. Different K values were examined to see the effect of VKR’s ancestral breeds in the population. In addition, admixture was run using all available individuals for each ancestral population versus a more balanced number of individuals to represent each population, thereby examining the influence of the number of individuals representing a breed on population structure. Reduced numbers of HOL and JER cattle were selected based on their breed purity through analysis against 9 other purebreds: Montbéliarde, Normande, Norwegian Red, Guernsey, Brown Swiss, Ayrshire, Braunvieh, Short Horn and Finnish Ayrshire (Upadhyay et al., 2019; Iso-Touru et al., 2016; Gautier et al., 2010) to provide a more comparable number of animals representing each ancestral breed. This analysis yielded 49 HOL and 44 JER animals with minimum 95% breed purity which were then included for admixture analysis with the admixed populations.

### 2.5 Comparison of genomic breed composition to performance traits

Phenotypic records were provided by Dairy Records Management Systems (Raleigh, NC) that included estimates of 305-days lactation records for Milk Yield (MY), Fat Yield (FY), Protein Yield (PY), and Somatic Cell Score (SCS). This dataset included more than 5,000 animals, including both admixed and parental populations used in the ProCROSS and Grazecross populations. However, only admixed animals with genotype data were considered and used for downstream analysis. The mean value of a trait was used for all animals with multiple recorded measurements. First, a predictive model was fit based on linear regression using linear model function in R version 4.1.1 (R Core Team, 2021). Models for each quantitative trait (Model ProCROSS and Model Grazecross) were analyzed using a base model, with covariate of genomic breed composition (GBC), breed generation (Gen) and sire breed (Sire), where the threshold for significance was considered at *p*-value < 0.05. Genomic breed composition (GBC) was obtained from the breed estimation produced from the Admixture analysis. Breed generation (Gen) was the generation number of the produced admix animals due to the rotational crossbreeding systems. Using this model, the effect of different explanatory variables on admixed animals’ performance was investigated.

Model ProCROSS=lm (Trait(s)∼ GBC+Gen+Sire )

Model Grazecross=lm (Trait(s) ∼ GBC+Gen+Sire )

Next, we evaluated the genomic breed composition of the animals between the two extreme tails in terms of performance to determine if there was a significant difference in the breed composition of animals related to performance. To achieve this, first the phenotypic data were processed in R version 4.1.1 (R Core Team, 2021) to determine normality and opposing extreme tails. Extreme tails in this study were defined by the top 20% as high-performance animals, while the lowest 20% were low-performance animals. The normality of each group was then determined using the Shapiro Test. The association of genomic breed composition from admixture analysis with extreme performance was conducted using a Student’s t-test function available in R (Chambers and Hastie, 1992). All associations between variables were assessed through the *p*-value produced by each test.

## 3 Result

### 3.1 Filtering and quality control of genomic data

The optimum number of informative SNP was identified by weighing various QC on the SNP common across genotyping platforms within the merged datasets. Since three different types of commercial bovine SNP chips were used for genotyping crossbred and reference animals in each dataset, we expected to see a reduced number of common SNP shared across platforms and passing QC (Wang et al., 2020). Indeed, only 15,708 SNP were common across the five commercial bovine SNP chips. Stringent quality parameters (MAF: >0.05, SNPs call rate: >95%, *r* ^*2*^ > 0.75) were selected to ensure no biasness in terms of the source of the SNPs; nevertheless, the number of SNPs should be sufficient to cover and represent the majority of regions across the genome and were successful in distinguishing population structure. Comparing genotyping call rates across all the SNP in both merged datasets implied that most of the samples had less than 0.3 call rate, which agreed with the total number of samples genotyped on 50K SNP (Illumina and Clarifide^©^) instead of HD150K (Illumina). Thus, SNPs with less than 95% call rate were excluded, resulting in the removal of 146,897 SNP for ProCROSS and 138,183 SNP for Grazecross. Next, 38 SNPs and 45 SNPs were excluded because minor alleles were less than 0.05, while 4 SNPs and 7 SNPs were dropped due to LD more than 0.75 with other SNP for ProCROSS and Grazecross datasets, respectively. In total, 9,792 SNPs and 10,795 SNPs were retained for ProCROSS and Grazecross datasets, respectively, for downstream analysis.

### 3.2 Principal component analysis

#### 3.2.1 Viking red representation

**FIGURE 1:**
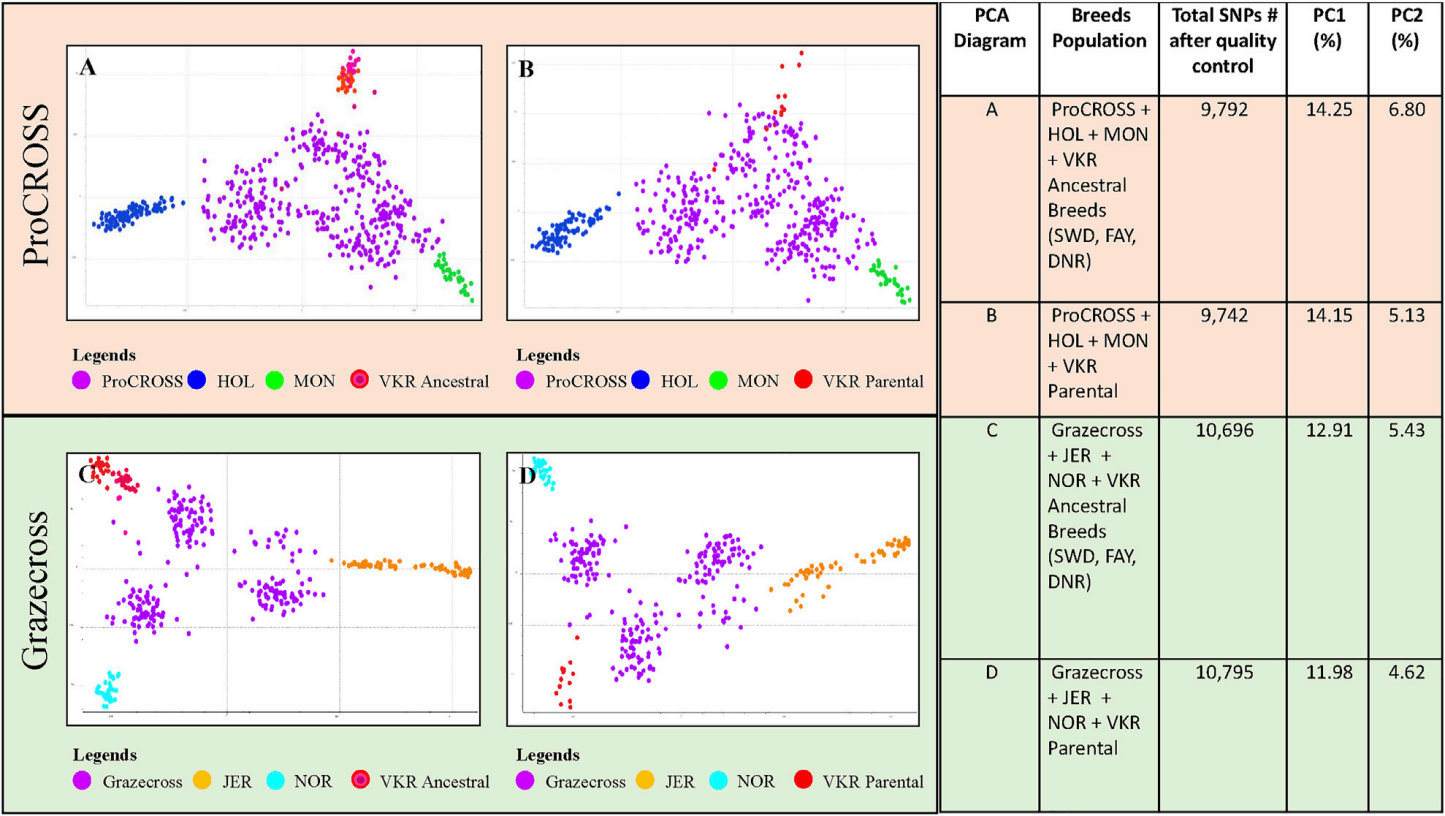
Principal component analysis comparing admixed populations of ProCROSS and Grazecross with their ancestral breeds. The top diagrams **(A,B)** depict ProCROSS with ancestral populations of HOL, MON, and VKR. The bottom diagrams **(C,D)** depict Grazecross with ancestral population of JER, NOR, and VKR. Left-hand diagrams **(A,C)** use the ancestral breeds of SWD, FAY and DNR to represent VKR. The right-hand diagrams **(B,D)** use commercial VKR sires used in the ProCROSS and Grazecross populations to represent VKR. The right-hand table shows the number of SNPs used for the PCA with the percentage of variation described by principal components 1 (*X*-axis) and 2 (*Y*-Axis) for each diagram.

Two VKR representations were assessed to investigate the effect of differing reference populations on breed composition and ancestry relationship to performance traits in the admixed populations. The PCA analysis of the VKR with its ancestral breeds (Supplementary Figure S2) showed a high level of similarity between populations with little substructure. In all, the VKR parental animals were spread along the *x*-axis, reflecting PC1 (1.94%) and among all of the ancestral breeds. Principal component 2 (0.97%) differentiated the DNR and one VKR parental animal from the others. Nonetheless, the different representations of VKR in the datasets provided different admixture measures that were used to correlate with the performance traits. The PCA plots in Figure 1 demonstrated the effect of two different VKR datasets on ProCROSS and Grazecross. The additional PCA analysis combining both VKR representations mirrored the same general clustering of the previous analyses (Supplementary Figure S3). All PCA were completed using different sets of autosomal SNPs identified after QC optimization, as listed in Figure 1.

Limited genetic sub-structure was revealed between populations in both admixed population PCA, further validated by the pairwise F_ST_ value (Table 2). In the ProCROSS population, the lowest genetic differentiation was observed between the two VKR representations, which is 0.0216 while the highest genetic differentiation was between HOL and JER with 0.1630. The same observation with the same population pair was shown in the Grazecross population with the lowest of 0.0218 and the highest of 0.1761, respectively. Comparing both VKR representations showed that parental VKR has a closer relationship with ProCROSS and Grazecross populations than ancestral VKR as expected.

**TABLE 2:** Pairwise genetic differentiation (F_ST_) value for all populations used in the ProCROSS and Grazecross breeding programs.

Population	ProCROSS	NOR	MON	JER	HOL	Ancestral VKR	Parental VKR
ProCROSS							
NOR	0.0706						
MON	0.0442	0.1158					
JER	0.0956	0.1350	0.1469				
HOL	0.0511	0.1272	0.1467	0.1630^a^			
Ancestral VKR	0.0375	0.0977	0.1143	0.1336	0.1131		
Parental VKR	0.0488	0.0938	0.1116	0.1267	0.1124	0.0216^b^	

**Population**	**Grazecross**	**NOR**	**MON**	**JER**	**HOL**	**Ancestral VKR**	**Parental VKR**
Grazecross							
NOR	0.0501						
MON	0.0826	0.1163					
JER	0.0605	0.1495	0.1617				
HOL	0.0881	0.1279	0.1474	0.1761^a^			
Ancestral VKR	0.0378	0.0981	0.1156	0.1510	0.1137		
Parental VKR	0.0463	0.0943	0.1123	0.1422	0.1135	0.0218^b^	

a The highest F_ST_, value within each admixed population.

b The lowest F_ST_, value within each admixed population.

#### 3.2.2 Population structure of ProCROSS and Grazecross populations

The PCA (Figure 1) for both populations revealed the expected distribution for each population cluster where the admixed population was at the center of the PCA while parental sources surrounded the admixed population in a triangle-like distribution for a three-crossbreed rotation. In the ProCROSS PCA, the highest component yielded 14.25% total variation, separating HOL and MON; the second component segregated the VKR population and yielded 6.58% total variation when using the ancestral VKR (Figure 1A) and 5.13% total variation when using the parental VKR (Figure 1B). Less variation was expected when comparing the parental VKR given their direct relationship to the ProCROSS. Some VKR individuals, regardless of which group representation, clustered with the ProCROSS, contributing to lower variation in the second component and demonstrating the higher degree of similarity between the VKR and the ProCROSS in general. In the Grazecross PCA, the highest component accounted for 12.91% of the total variation, separating JER and NOR; the second component accounted for 5.43% variation using the ancestral VKR (Figure 1C) and 4.62% variation using the parental VKR (Figure 1D), with a clear separation between VKR and Grazecross. Overall, similar PCA clustering can be observed in the ProCROSS and Grazecross PCA with clear sub-clustering of the admixed populations, especially in Grazecross. Since both admixed populations were developed from continuous rotational crossbreeding, the admixed individuals sub-cluster based on the most recent breed of sire (Supplementary Figure S4). The dominant ancestry or breed is determined by the most recent purebred sire from the 3 major breeds. This is because, without considering the generation number to produce the admixed animals, the most recent purebred sire will contribute at least 50% to the offspring’s breed composition.

### 3.3 Breed composition

#### 3.3.1 Pedigree based

**FIGURE 2:**
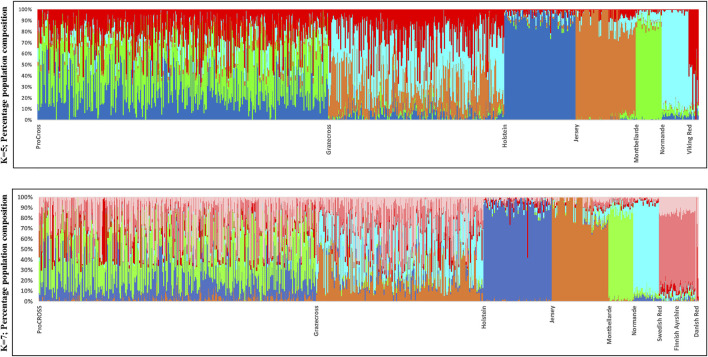
Admixture analysis reflecting genetic clustering of breeds for both ProCROSS and Grazecross admixed and all parental populations for *K* = 5 and *K* = 7. Individual vertical bars along the *X*-axis represent individual cattle grouped by breed; *Y*-axis provides a measure of the composition of each genetic population found within individuals.

**FIGURE 3:**
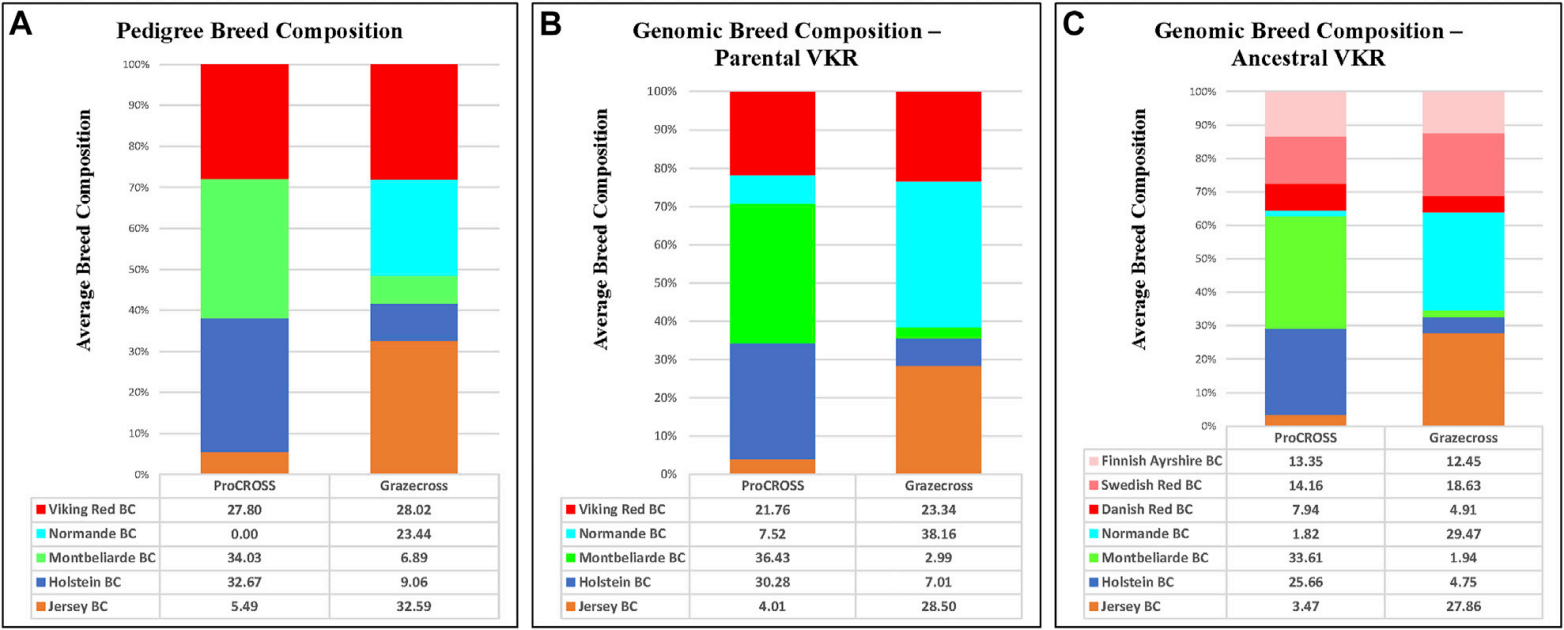
Average breed composition using pedigree or genomic data; parental or ancestral VKR representation. **(A)** Pedigree based breed composition **(B)** Genomic based breed composition with commercial VKR as reference panel **(C)** Genomic based breed composition with ancestral VKR as reference panel.

In general, both ProCROSS and Grazecross admixed animals have proportions of ancestral breeds between 23.4% and 34%, which was expected with the 3 breeds crossbreeding system (Figures 2, 3). Ideally, three subgroup signatures were considered to associate with the typical ancestry sources in each admixed population. However, nontypical breeds were identified in some of the admixed individuals based on pedigree information. For example, JER is not a typical breed component in ProCROSS populations, while HOL and MON are atypical in Grazecross. Due to that reason, a slightly lower estimation of the major breeds in the Grazecross population was due to two nontypical breeds detected at marginally higher levels in the pedigree information. A higher HOL breed composition of 9.0% was estimated because the foundation animal of the Grazecross population is purebred HOL (Pereira and Heins, 2019). Recent sire breed plays a major role in determining admixed individual’s breed composition. Due to the same effect of foundation animal, ProCROSS admixed animals with HOL as the recent sire breed were estimated to have the HOL breed proportion between 57.8% and 62.5%, while the other two recent sire sources of MON and VKR were estimated between 50.0% and 57.0%.

### 3.3.2 Genomic based breed composition with admixture

Admixture results were used to estimate genomic breed composition for ProCROSS and Grazecross individuals. Admixture analysis was run with *K* = 5 and *K* = 7 in an unsupervised clustering to assess overall population structure, comparing ProCROSS or Grazecross and their ancestral populations, again, interchanging representation of the VKR group as shown in Figure 2 (Top) was the result from admixture analysis *K* = 5 where VKR were the commercial parental source animals, while Figure 2 (Bottom) used VKR’s ancestral breeds. A clear distinction could be seen for most purebred populations, which shows greater homogeneity and less admixture within the HOL, JER, MON, and NOR. In contrast, the SWD and FAY shared the same genetic signature, excluding the DNR breed signature representation.

In slight contrast, in the admixture analysis (Supplementary Figure S3) combining both VKR and its ancestral populations along with all other breeds and both admixed populations, a higher degree of admixture was observed within individuals of the commercial VKR, SWD, and FAY, whereas DNR still produced its own unique genetic signature. Using this same combined dataset of all individuals, admixture results were used to generate average genomic-based breed composition (Figure 3). All nontypical breeds in both admixed populations were detected at low levels within the admixed individuals. Depending on which VKR datasets were used, JER composition was estimated between 3.5 and 4.0% in ProCROSS, while MON ranged from 2.0% to 3.0% in Grazecross. However, a slightly higher nontypical HOL composition was detected in Grazecross, ranging between 4.8 and 7.0%. These results coincide with expectations of all the admixed individuals used in both programs originating from HOL (Pereira and Heins, 2019) and pedigree breed composition estimates. Figure 3 also suggests that the usage of ancestral VKR as the parental panel may overestimate the VKR composition in the admixed population. Collectively, ancestral VKR representation is estimated to contribute 35.5 and 36% in ProCROSS and Grazecross, respectively. This estimation is significantly different (*p*-value < 0.05) from the estimation produced from using the commercial VKR as the parental panel, which is 21.8 and 23.3% for ProCROSS and Grazecross populations. This difference in VKR composition influenced the estimation of other breed compositions. Except for Jersey composition in Grazecross, the *t*-test analysis revealed significant differences (*p*-value < 0.05) in all breed composition estimates between the two VKR representation datasets (Figure 3).

**FIGURE 4:**
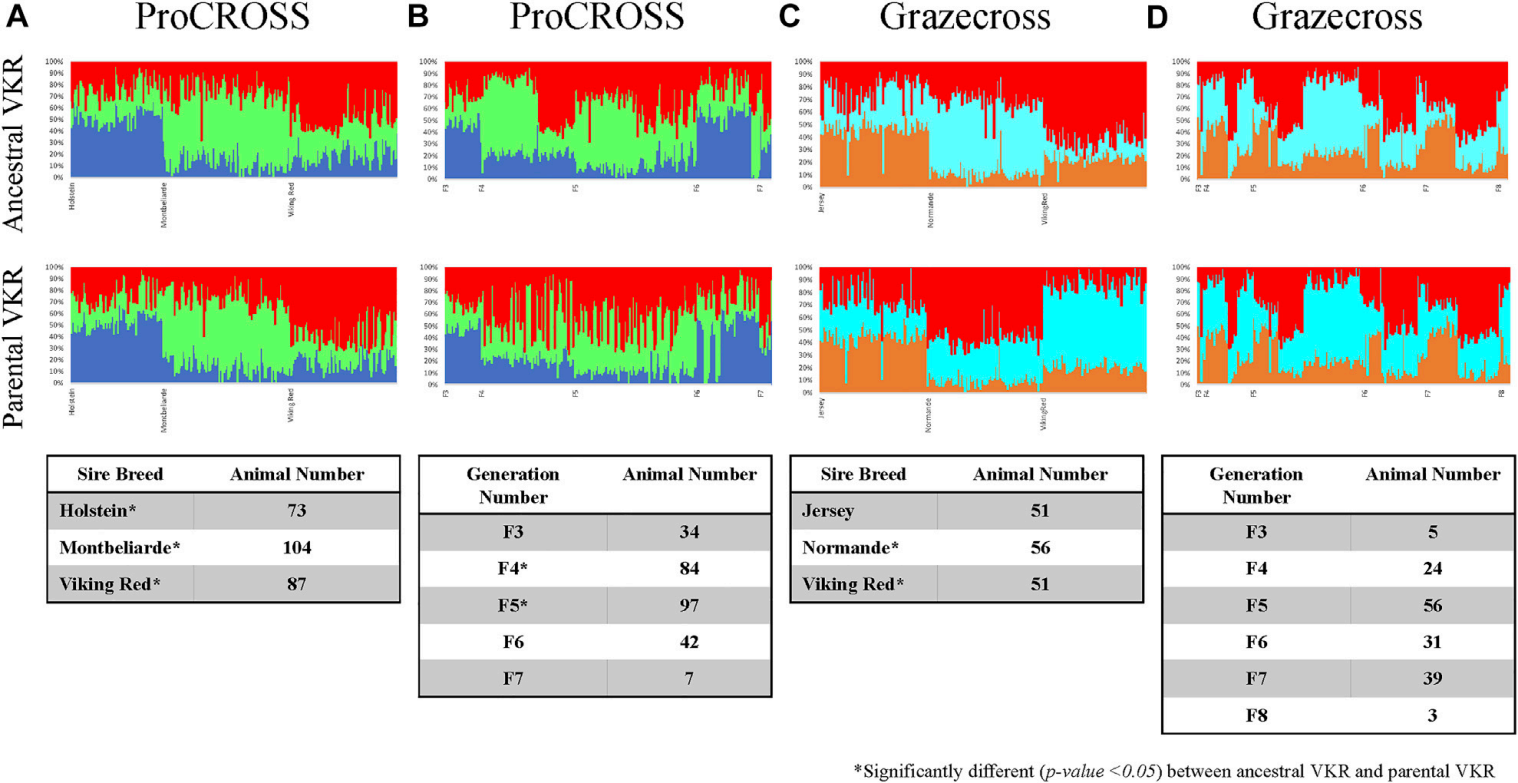
Admixture analysis for ProCROSS and Grazecross individuals using all major parental populations at *K* = 3. The top row used ancestral VKR (FAY, DNR, and SWR) as the reference population whereas the bottom row used parental VKR as the reference population. Columns **(A,C)** ordered admixed individuals across the *X*-axis based on their recent sire while columns **(B,D)** ordered animals based on the generation of birth to better view the impact of these variables on the admixed populations. Tables within each column describe the distribution of admixed individuals based on assigned variables.

The same observation could also be seen in genomic breed composition, in which the recent sire breed has the most extensive composition in the admixed population. The magnitude of the effect of the sire breed was analyzed in ADMIXTURE software. Figure 4 was the admixture plot produced based on *K* = 3, representing the three main breeds in each admixed population ordered based on recent sire breeds (Figures 4A,C) and breed generation information (Figures 4B,D). The admixture plots arranged based on sire breeds showed three genetic patterns across both the admixed populations as followed in Supplementary Figure S4. Factoring in the VKR representation showed a significant difference in the major breed composition estimation except for JER. The rotational systems used for these research herds produced a mixed individual developed from multiple generations of crossbreeding. The ProCROSS population consisted of offspring from the F3 generation up to the F7 generation while the Grazecross population consisted of offspring from the F3 generation up to the F8 generation. Despite this, very little sub-clustering was observed based on order by generation as opposed to order by recent sire. This result may have been confounded by some of the admixed individuals in this dataset undergoing different rotations of sire breed (for example, HOL-MON-VKR versus HOL-VKR-MON) for the purpose of other research objectives. Still, a comparison of the breeds estimation produced between the two VKR representations showed a small effect on the breed estimation when considering generation where F4 and F5 individuals in ProCROSS showed a significant difference. No significant difference in breed composition was observed in the Grazecross individuals in the aspect of generation number.

The reliability of pedigree-estimated breed composition can be compromised by missing, inaccurate, or incomplete records (Akanno et al., 2018). The pedigree composition was compared with estimates of genomic breed composition produced from the Q matrix in unsupervised mode; genomic breed composition offers more robust result estimations (Chiang et al., 2010). Factors such as a study population composed of only unrelated animals, adequate representation of all ancestral breeds, and low levels of linkage equilibrium between markers improve genomic breed-composition accuracy produced in the Q matrix (Alexander et al., 2009). Therefore, pedigree and genomic breed estimations were compared across admixed individuals using the two different VKR representations.

**FIGURE 5:**
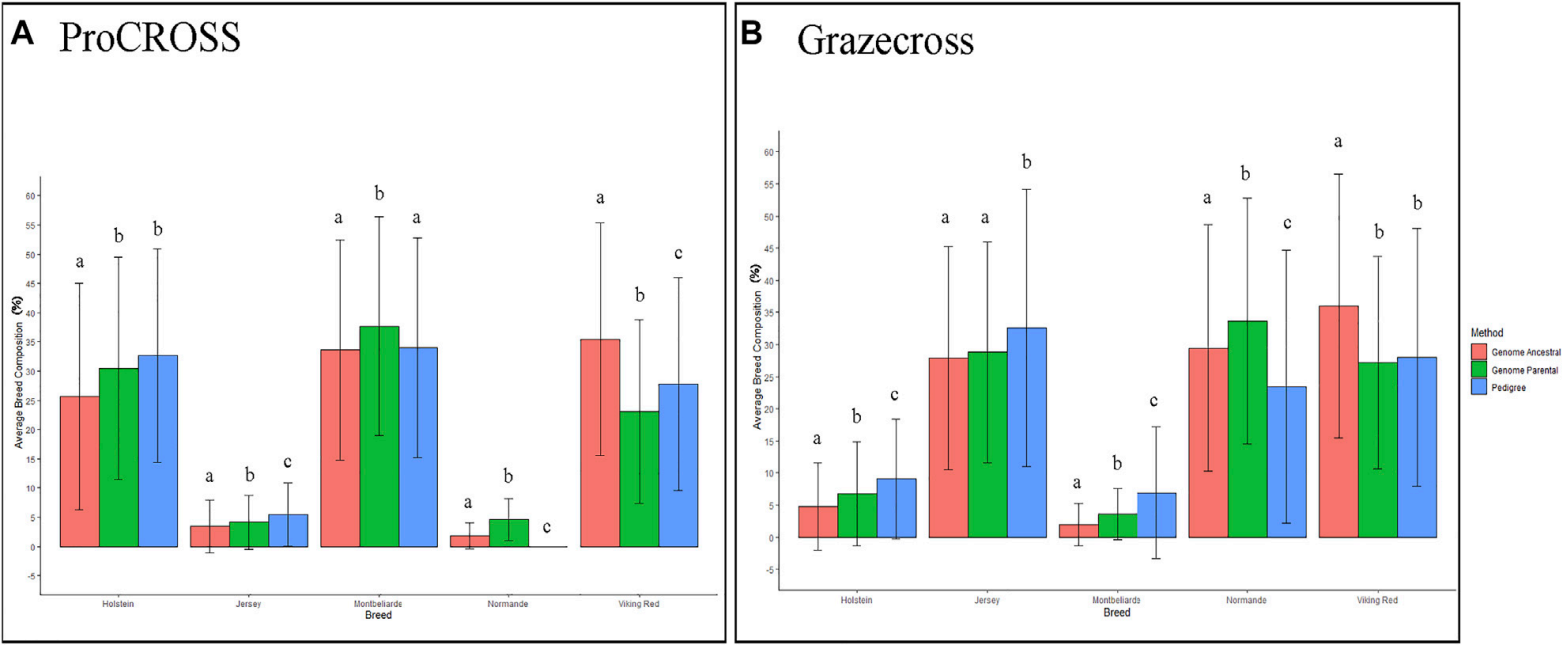
Comparison of average breed composition based on pedigree and genomic breed estimations comparing VKR population influence. Barplot A is for ProCROSS datasets and Barplot B is for Grazecross dataset. *X*-axis represent the average breed composition; *Y*-axis provides the percentage of breed composition. A *t*-test is used to compare between the methods/datasets the change in breed composition average. Error bars use one standard deviation and letters indicate significant difference between estimations with *p*-value < 0.05 within breed composition.

Figure 5 categorizes the variation in breed estimations for each pure breed used in the analysis, distinguishing pedigree, VKR parental, and VKR ancestral populations. For every breed found in ProCROSS or Grazecross, except for JER in Grazecross, there was a significant difference (*p*-value < 0.05) in average breed estimation dependent upon the use of either the ancestral VKR or the parental VKR. However, results were not as straightforward when comparing the two genomic estimates to pedigree estimates, with sometimes one or the other VKR representation causing changes in estimates or being more similar to pedigree estimates. In ProCROSS, the use of ancestral VKR resulted in a comparable MON composition but reduced the HOL composition and increased the VKR composition as compared to the pedigree estimation. In Grazecross, ancestral VKR also caused an increase in both VKR and NOR composition while decreasing the JER composition based on the *p*-value produced from the *t*-test. On the other hand, the commercial parental VKR estimated a closer HOL and VKR composition with the pedigree estimation in ProCROSS and Grazecross populations. The smaller datasets consisting of select HOL and JER animals that demonstrated >95% breed purity did not yield significant differences (*p*-value < 0.05) in breed estimations for the admixed individuals.

### 3.4 Phenotypic association with breed composition

The effect of recent sire breed and breed generation was assessed with the genomic breed composition. Following the PCA plot in Supplementary Figure S4, a clear sub-cluster was shown in both admixed populations. This sub-cluster can also be seen in the admixture plot of Figure 4 when the admixed individuals are ordered based on their recent sire breed. The overall plot for the four (4) major traits MY, FY, PY, and SCS versus these two variables (Supplementary Figure S5) suggested a linear relationship between them, particularly for breed recent sire. Higher MY, FY, and PY ProCROSS cattle tend to have HOL as the recent sire, whereas Grazecross cattle with lower SCS have VKR as their recent sire. In assessing breed generation, most cattle in both admixed populations with the smallest number of generations (F3) tend to produce animals with higher performance in MY, FY, and PY traits than other generations.

Both linear regression models supported the observed trends that recent breed sire has a significant effect on performance traits (*p*-value < 0.05) including all three major breeds for MY, FY, and PY performance in the ProCROSS population and SCS performance in Grazecross (Supplementary Figure S4). However, the generation of the admixed animal (F3, F4, F5, F6, etc) typically did not significantly affect trait performance. Only the F6 generation of ProCROSS individuals showed an association to higher performance in contrast to the F3 generation that seemed to carry this trend in the plot. Both models successfully explained a high number of variance with a R-squared value between 93.1 and 97.4%.

The *t*-test analysis investigated whether breed composition is significantly different in high versus low-performance groups, confirming that breed composition is indeed important to performance. Fifty-two admixed individuals in each of the two extreme tails were selected in the ProCROSS population, while 32 individuals represented each extreme group in the Grazecross population. Then, the *t*-test was used to evaluate the difference in average genomic breed composition using the two VKR representations. The *t*-test results (Table 3) suggested that the percentage of MON and HOL composition plays a significant role in MY, FY, and PY in ProCROSS, whereas VKR and NOR composition plays a significant role in Grazecross SCS. Despite the degree of certain breeds being significantly different within elite and poor performing ProCROSS and Grazecross for specific traits, there was a substantial degree of variation in breed composition within each performance group. For instance, in the high-performance ProCROSS population, the HOL composition ranged between 9.0 and 68.0%, whereas MON composition ranged between 10.0 and 75.0% in individuals. On the other hand, low-performance animals were estimated to have 3.0–68.0% HOL composition and 4.0–75.0% MON composition. The same amount of variation could be seen in the Grazecross population. High-performance Grazecross individuals were estimated to have between 3.0 and 56.0% NOR or 26.0%–78.0% VKR composition. While low-performance individuals had a similar degree of variation of 5.0%–68.0% NOR or 10.3%–80.0% VKR composition. Despite many of the previous analyses showing that VKR representation had a significant effect on breed estimation, the usage of different VKR representations did not show an effect on determining performance association with the breed composition. Both VKR representations yielded the same breeds corresponding to different traits’ performance in both admixed populations, with the same ancestries being significantly associated with performance.

**TABLE 3:** p-value of comparison between 20% high performance and 20% low-Performance group in both admixed populations with different VKR’s datasets.

	Ancestral VKR					Parental VKR				
ProCROSS	Trait	Mean performance difference	P(T ≤ t) two-tail			Trait	Mean performance difference	P(T ≤ t) two-tail		
			HOL BC	MON BC	VKR BC			HOL BC	MON BC	VKR BC
	Milk yield	7428.96 (lb)*	0.00049*	0.00311*	0.73512	Milk yield	7428.96 (lb)*	0.00026*	0.01237*	0.41987
	Fat yield	266.86 (lb)*	0.00011*	0.00012*	0.87084	Fat yield	266.86 (lb)*	0.00008*	0.00025*	0.96101
	Protein yield	239.76 (lb)*	0.00155*	0.00263*	0.96903	Protein yield	239.76 (lb)*	0.00100*	0.00785*	0.74218
	Somatic cell score	2.93*	0.52640	0.35307	0.71494	Somatic cell score	2.93*	0.29760	0.78010	0.44794

Grazecross	Trait	Mean performance difference	P(T ≤ t) two-tail			Trait	Mean performance difference	P(T ≤ t) two-tail			
			JER BC	Nor BC	VKR BC			JER BC	Nor BC		VKR BC
	Milk yield	6609.60 (lb)*	0.31386	0.70962	0.58421	Milk yield	6609.60 (lb)*	0.35468	0.50522		0.90795
	Fat yield	253.75 (lb)*	0.22590	0.64534	0.10836	Fat yield	253.75 (lb)*	0.21537	0.73295		0.13973
	Protein yield	217.50 (lb)*	0.42124	0.85183	0.57467	Protein yield	217.50 (lb)*	0.45279	0.67317		0.82319
	Somatic cell score	2.96*	0.52466	0.00060*	0.00325*	Somatic cell score	2.96*	0.61365	0.00090*		0.00231*

Significant difference with *p*-value < 0.05.

## 4 Discussion

The PCA plots in Figure 1 provided the first insight into the genomic population structure of the admixed ProCROSS and Grazecross and their three major parental breeds. Distinct separation was seen between the breeds and the admixed populations except for an overlap between VKR representation and the ProCROSS. In general, there was a greater distinction between the ancestral VKR and the ProCROSS and more overlap between the parental VKR and ProCROSS, as expected given the difference in relationship to the ProCROSS progeny. Even though VKR is the marketing term for the breed that share the same ancestry, such as SWD, FAY, DNR, and even Norwegian Red. Our selected SNPs successfully showed to differentiate these two VKR representations. The observation was supported by F_ST_ calculated in Table 2; both ProCROSS and Grazecross showed a comparable genetic differentiation from parental VKR at 0.038, which is lower than the F_ST_ estimated using ancestral VKR (0.049 and 0.046 respectively). Although the number of individuals used to represent both VKR datasets was imbalanced, estimated F_ST_ provide intrinsic evidence of the sufficient power of the number of informative SNPs used in the analysis to discriminate between all the populations, including both VKR representations. The 229 Grazecross animals showed a higher degree of separation within their population, with 3 sub-groups reflecting recent sires. While the 378 ProCROSS had the same basic sub-structure reflecting recent sire used, there was less separation between the 3 sub-groups. This slight variation in the degree of sub-structure may result from 17 unique sires used in developing the larger population of ProCROSS animals compared to 12 sires used in the development of the smaller Grazecross population. Other considerations may be due to the ProCROSS breeds being more related than the Grazecross breeds due to the effect of their parental source. VKR is not purebred with historical crossbreeding, which may include HOL (Dechow and Hansen, 2017). In addition, Red and white HOL have been used historically in MON improvement (Heins et al., 2006a; Heins et al., 2006b). On the contrary, Grazecross parental NOR is unlikely to have contributed to the development of VKR, and JER was mostly pure until recently (Dechow and Hansen, 2017).

Due to the genomic similarities between breeds or signatures of breed mixture within individuals, the estimated genomic breed composition for purebred animals is complicated by small admixture components. Thus, not all animals designated as purebred have 100% genomic breed composition for their respective breed categories, as shown in Figure 2. This increases complexity in estimating accurate breed composition in an admixed population. The purebred populations used in this analysis included HOL, JER, MON, and NOR. The animals representing each breed had an average breed composition of 92, 90, 86, and 87%, respectively. In comparing different representative purebred animals having differing purebred breed averages, no significant effect on the admixed population breed composition estimation was seen. These findings support our assumption that any changes in the breed composition estimation are due to the different sources of the VKR population used in the analysis.

With the exception of the JER breed composition in Grazecross, all other estimates of the purebred contribution to the ProCROSS and Grazecross showed a significant difference in their estimation when using the VKR ancestral versus the VKR parental (Figure 5) groups. Subsequently, both estimations were compared to the estimation produced from pedigree information. It is noted that in Figure 3, the use of VKR parental animals in ProCROSS increased the MON origin to 36.4%, compared to the pedigree estimation of 34.0% which was a significant difference between the estimations. This could be explained by VKR and MON being selected for the same fertility and health traits for over 30 years (Heringstad et al., 2007; Dezetter et al., 2015). This observation may prove that both breeds shared comparable allele frequencies due to similar selection pressure on the same haplotype, causing increased similarity between these two breeds. Whereas the use of VKR parental in Grazecross also significantly increased NOR composition. Hence, including VKR ancestral reference breeds introduced a higher noise level in estimating purebred composition (Figure 2), offsetting the estimated composition for the ancestral breeds. The rest of the estimated genomic breed composition using the VKR ancestral datasets showed similar patterns. Except for VKR estimations, using the ancestral VKR representation decreased other breed composition within admixed individuals compared to both pedigree and genomic estimates using the parental VKR representation.

Next, the correlation between genomic breed composition and four traits was investigated. Both linear regression models suggested that all three major breeds significantly affect the MY, FY, and PY production traits in ProCROSS and health trait SCS trait in Grazecross as denoted in Supplementary Figure S5. This observation was further explored by comparing the extreme performance groups of each trait. Surprisingly, the results shown in Table 3 indicate no significant difference in ancestry importance for performance when comparing the two sources of VKR (*p*-value < 0.05). Both VKR representations identified the same breeds as being significantly different between performance groups and at similar levels within the elite or poor performance groups. As expected, HOL and MON composition were significantly higher in the animals with higher performance in MY, FY, and PY. HOL is known to have superior performance in milk yield, whereas MON was improved for milk solids to produce speciality cheese, resulting in a higher emphasis on the fat and protein yield traits. Thus, both of these breeds contribute to the high production performance of the admixed population. In contrast, in Grazecross, only NOR and VKR showed a significant difference in composition related to SCS. The Grazecross admixed population was developed to cater to demand for efficient high performing animals in low input grazing environments. Thus, different traits and breeds were used for this admixed population. NOR was developed in Northwestern France, a region known to have pasture. This environment produced a breed with exceptional feed conversion rates, making it a great genetic source for developing low grazing crossbred cattle (Dillon et al., 2003). The same study showed that NOR has a substantially higher survival rate than three dairy cattle breeds, including Dutch Holstein, Irish Holstein, and Montbeliarde. In addition, VKR was developed to have higher fertility and health performance (Heringstad et al., 2007). This complements our finding that high-performance Grazecross animals in SCS have a higher NOR and VKR composition. However, we did not see the same complementary effect on SCS performance in ProCROSS with regards to their VKR ancestry. One thought potentially explaining this variation in VKR correlation to SCS is the influence of JER ancestry within Grazecross that is not present in ProCROSS. The JER breed is known to have higher SCS compared to other dairy breeds in the USA (Dechow and Hansen, 2017). Thus, as NOR and VKR breed proportions increase in Grazecross, the JER breed proportion is decreasing which may contribute to the reduction in SCS even though JER were not statistically associated with this trait. It may be the unique combination of the different breeds used in each of these 3-breed rotations that alters the degree of impact of any breed on a performance trait. Furthermore, individually assessing the animals within the two extreme groups showed a dispersed distribution of the identified breeds. For instance, higher HOL composition cattle have the highest chance of producing high-performance production animals, yet some elite production animals only had 4% HOL composition and vice versa in the low-performance animals. These observations may suggest that some haplotypes at specific locations have a large effect on determining the performance of these traits. For instance, the elite 4% HOL ProCROSS suggests that they possess HOL ancestry at key genomic regions most influential on production and additional HOL ancestry is not actually required to be elite. Therefore, the association between increased performance and increased HOL breed ancestry could be based on the fact that increased HOL ancestry increases the chance that an animal possesses HOL ancestry at the most important regions of the genome as seen in the 4% HOL ProCROSS and the remainder of the HOL ancestry is incidental to their performance. Key haplotypes may be directly linked and preserved in particular ancestral breeds used in the crossbreeding program. Identifying the existence and influence of breed-specific haplotypes in future studies may leverage breed complementary and accelerate selection through more precise and accurate genomic prediction. In all, the correlation analysis was also able to capture the effect of heterosis on the performance of both admixed populations. Interestingly, the linear regression model suggested that individuals developed from generation six (two round of 3 breed rotational crossbreeding systems) significantly capitalize on heterosis similar with what we expected from generation three (One round of 3 breed rotational crossbreeding systems). This observation may serve as additional evidence of the advantages provided by a 3-breed rotational crossbreeding system to retain the highest heterosis level in the crossbreeding population.

## 5 Conclusion

The main inquiry of this project was to identify the impact of different animal groups serving as the reference population when assessing admixture in crossbred cattle. The results showed differences in the estimated breed composition of both ProCROSS and Grazecross individuals when two different VKR reference panels were used. Interestingly, the breed composition estimates from admixture were significantly different from pedigree estimates but the variation sometimes diverged, with one panel having an increased estimate of a particular breed when the other reference panel would show a lower estimate of that same breed as compared to the pedigree estimate. Surprisingly, despite the two different VKR references panels providing significantly different breed compositions of ProCROSS and Grazecross, we found no significant difference in the relationship of breed composition to performance traits as HOL, MON, NOR, and VKR were all similarly important to ProCROSS and Grazecross performance. It is important to identify the most appropriate and informative animals to use as reference animals in admixture analysis to correctly interpret relationship and population structure results. Any application of admixture output in directing dairy cattle crossbreeding strategies should proceed with caution depending upon the reference populations used to prevent over- or under-interpretation of the contribution and impact of ancestry breeds.

## Data Availability

1) The data presented in the study are deposited in the University of Minnesota Digital Conservancy repository, https://hdl.handle.net/11299/227134; 2) The data is uploaded and restricted for 2 years, which means if people want to download, they have to send the data owner’s (Bradly J. Heins) an email to request access. The data will open access after the 2 years per UM.

## References

[B1] Akanno E. C. Abo-Ismail M. K. Chen L. Crowley J. J. Wang Z. Li C. (2018). Modeling heterotic effects in beef cattle using genome-wide SNP-marker genotypes1. J. Anim. Sci. 96 (3), 830–845. 10.1093/jas/skx002 29373745 PMC6093554

[B2] Alexander D. H. Novembre J. Lange K. (2009). Fast model-based estimation of ancestry in unrelated individuals. Genome Res. 19 (9), 1655–1664. 10.1101/gr.094052.109 19648217 PMC2752134

[B3] Berry D. P. (2018). Symposium review: Breeding a better cow-Will she be adaptable? J. Dairy Sci. 101 (4), 3665–3685. 10.3168/jds.2017-13309 29224864

[B4] Berry D. P. Wall E. Pryce J. E. (2014). Genetics and genomics of reproductive performance in dairy and beef cattle. animal 8 (1), 105–121. 10.1017/S1751731114000743 24703258

[B5] Chang C. Chow C. Tellier L. Vattikuti S. Purcell S. M. Lee J. (2015). Second-generation PLINK: Rising to the challenge of larger and richer datasets. GigaSci 4, 7–015. 10.1186/s13742-015-0047-8 PMC4342193 25722852

[B6] Chiang C. W. Gajdos Z. K. Korn J. M. Kuruvilla F. G. Butler J. L. Hackett R. (2010). Rapid assessment of genetic ancestry in populations of unknown origin by genome-wide genotyping of pooled samples. PLoS Genet. 6, e1000866. 10.1371/journal.pgen.1000866 20221249 PMC2832667

[B8] Dechow C. D. Hansen L. B. (2017). “Capitalizing on breed differences and heterosis,” in Large dairy herd management. 3rd ed (Champaign: American Dairy Science Association), 369–378. 10.3168/ldhm.0526

[B9] Dezetter C. Leclerc H. Mattalia S. Barbat A. Boichard D. Ducrocq V. (2015). Inbreeding and crossbreeding parameters for production and fertility traits in Holstein, Montbéliarde, and Normande cows. J. Dairy Sci. 98 (7), 4904–4913. 10.3168/jds.2014-8386 25981069

[B10] Dillon P. Buckley F. O’Connor P. Hegarty D. Rath M. (2003). A comparison of different dairy cow breeds on a seasonal grass-based system of milk production. Livest. Prod. Sci. 83 (1), 21–33. 10.1016/s0301-6226(03)00041-1

[B11] Gautason E. Schönherz A. A. Sahana G. Guldbrandtsen B. (2021). Genomic inbreeding and selection signatures in the local dairy breed Icelandic Cattle. Anim. Genet. 52 (3), 251–262. 10.1111/age.13058 33829515

[B12] Gautier M. Laloë D. Moazami-Goudarzi K. (2010). Insights into the genetic history of French cattle from dense SNP data on 47 worldwide breeds. PloS one 5, e13038. 10.1371/journal.pone.0013038 20927341 PMC2948016

[B13] Gurgul A. Szmatoła T. Topolski P. Jasielczuk I. Żukowski K. Bugno-Poniewierska M. (2016). The use of runs of homozygosity for estimation of recent inbreeding in Holstein cattle. J. Appl. Genet. 57 (4), 527–530. 10.1007/s13353-016-0337-6 26803654

[B14] Heins B. J. (2019). “Opportunities and challenges in crossbreeding dairy cattle in temperate regions” in Pryce, J., Advances in breeding of dairy cattle (Cambridge, UK: Burleigh Dodds Science Publishing). 10.19103/as.2019.0058.06

[B15] Heins B. J. Hansen L. B. De Vries A. De Vries A. (2012). Survival, lifetime production, and profitability of Normande × Holstein, Montbéliarde × Holstein, and scandinavian red × Holstein crossbreds versus pure holsteins. J. Dairy Sci. 95 (2), 1011–1021. 10.3168/jds.2011-4525 22281364

[B16] Heins B. J. Hansen L. B. Hazel A. R. Seykora A. J. Johnson D. G. Linn J. G. (2010). Birth traits of pure Holstein calves versus Montbeliarde-sired crossbred calves. J. Dairy Sci. 93 (5), 2293–2299. 10.3168/jds.2009-2911 20412946

[B17] Heins B. J. Hansen L. B. Seykora A. J. (2006b). Calving difficulty and stillbirths of pure holsteins versus crossbreds of Holstein with Normande, Montbeliarde, and scandinavian red. J. Dairy Sci. 89 (7), 2805–2810. 10.3168/jds.S0022-0302(06)72357-8 16772600

[B18] Heins B. J. Hansen L. B. Seykora A. J. (2006a). Production of pure holsteins versus crossbreds of Holstein with Normande, Montbeliarde, and scandinavian red. J. Dairy Sci. 89 (7), 2799–2804. 10.3168/jds.S0022-0302(06)72356-6 16772599

[B19] Heins B. J. Hansen L. B. Seykora A. J. Hazel A. R. Johnson D. G. Linn J. G. (2008b). Crossbreds of Jersey × Holstein compared with pure holsteins for body weight, body condition score, dry matter intake, and feed efficiency during the first one hundred fifty days of first lactation. J. Dairy Sci. 91 (9), 3716–3722. 10.3168/jds.2008-1094 18765631

[B20] Heins B. J. Hansen L. B. Seykora A. J. Hazel A. R. Johnson D. G. Linn J. G. (2011). Short communication: Jersey × Holstein crossbreds compared with pure holsteins for production, mastitis, and body measurements during the first 3 lactations. J. Dairy Sci. 94 (1), 501–506. 10.3168/jds.2010-3232 21183062

[B21] Heins B. J. Hansen L. B. Seykora A. J. Johnson D. G. Linn J. G. Romano J. E. (2008a). Crossbreds of Jersey × Holstein compared with pure holsteins for production, fertility, and body and udder measurements during first lactation. J. Dairy Sci. 91 (3), 1270–1278. 10.3168/jds.2007-0564 18292285

[B22] Heringstad B. Klemetsdal G. Steine T. (2007). Selection responses for disease resistance in two selection experiments with Norwegian red cows. J. Dairy Sci. 90 (5), 2419–2426. 10.3168/jds.2006-805 17430946

[B23] Iso-Touru T. Tapio M. Vilkki J. Kiseleva T. Ammosov I. Ivanova Z. (2016). Genetic diversity and genomic signatures of selection among cattle breeds from Siberia, eastern and northern Europe. Anim. Genet. 47 (6), 647–657. 10.1111/age.12473 27629771

[B24] Leroy G. Baumung R. Boettcher P. Scherf B. Hoffmann I. (2016). Review: Sustainability of crossbreeding in developing countries; definitely not like crossing a meadow. Animal 10 (2), 262–273. 10.1017/s175173111500213x 26503101

[B25] Mbole-Kariuki M. N. Sonstegard T. Orth A. Thumbi S. M. Bronsvoort B. D. C. Kiara H. (2014). Genome-wide analysis reveals the ancient and recent admixture history of East African Shorthorn Zebu from Western Kenya. Heredity 113 (4), 297–305. 10.1038/hdy.2014.31 24736786 PMC4181064

[B26] McAllister A. J. Lee A. J. Batra T. R. Lin C. Y. Roy G. L. Vesely J. A. (1994). The influence of additive and nonadditive gene action on lifetime yields and profitability of dairy cattle. J. Dairy Sci. 77 (8), 2400–2414. 10.3168/jds.S0022-0302(94)77183-6 7962862

[B27] Pereira G. M. Heins B. J. (2019). Activity and rumination of Holstein and crossbred cows in an organic grazing and low-input conventional dairy herd. Transl. Animal Sci. 3 (4), 1435–1445. 10.1093/tas/txz106 PMC7200548 32704908

[B28] Pryce J. E. Haile-Mariam M. Goddard M. E. Hayes B. J. (2014). Identification of genomic regions associated with inbreeding depression in Holstein and Jersey dairy cattle. Genet. Sel. Evol. 46 (1), 71–14. 10.1186/s12711-014-0071-7 25407532 PMC4234836

[B29] R Core Team (2021). R: A language and environment for statistical computing. R Found. Stat. Comput. Vienna.Austria. *URL* .

[B30] Stella A. Ajmone-Marsan P. Lazzari B. Boettcher P. (2010). Identification of selection signatures in cattle breeds selected for dairy production. Genetics 185 (4), 1451–1461. 10.1534/genetics.110.116111 20479146 PMC2927769

[B31] Touchberry R. W. (1992). Crossbreeding effects in dairy cattle: The Illinois experiment, 1949 to 1969. J. Dairy Sci. 75 (2), 640–667. 10.3168/jds.s0022-0302(92)77801-1 1560149

[B32] Upadhyay M. Eriksson S. Mikko S. Strandberg E. Stålhammar H. Groenen M. A. (2019). Genomic relatedness and diversity of Swedish native cattle breeds. Genet. Sel. Evol. 51 (1), 56–11. 10.1186/s12711-019-0496-0 31578144 PMC6775670

[B33] VanRaden P. M. (2017). “Genomic tools to improve progress and preserve variation for future generations,” in Book of abstracts of the 68th annual meeting of the European federation of animal science. (Vol. 79).

[B34] VanRaden P. M. Sanders A. H. (2003). Economic merit of crossbred and purebred US dairy cattle. J. Dairy Sci. 86 (3), 1036–1044. 10.3168/jds.S0022-0302(03)73687-X 12703641

[B35] Wang Y. Wu X. L. Li Z. Bao Z. Tait R. G. Jr Bauck S. (2020). Estimation of genomic breed composition for purebred and crossbred animals using sparsely regularized admixture models. Front. Genet. 11, 576. 10.3389/fgene.2020.00576 32595700 PMC7300184

[B36] Zhao F. McParland S. Kearney F. Du L. Berry D. P. (2015). Detection of selection signatures in dairy and beef cattle using high-density genomic information. Genet. Sel. Evol. 47 (1), 49–12. 10.1186/s12711-015-0127-3 26089079 PMC4472243

[B37] Zimin A. V. Delcher A. L. Florea L. Kelley D. R. Schatz M. C. Puiu D. (2009). A whole-genome assembly of the domestic cow, Bos taurus. Genome Biol. 10 (4), R42–R10. 10.1186/gb-2009-10-4-r42 19393038 PMC2688933

