# Supplementary figures and images for: The impact of using different ancestral reference populations in assessing crossbred population admixture and influence on performance
Source: Front Genet. 2022 Sep 26;13:910998. doi: 10.3389/fgene.2022.910998 (PMC9549382; doi:10.3389/fgene.2022.910998)

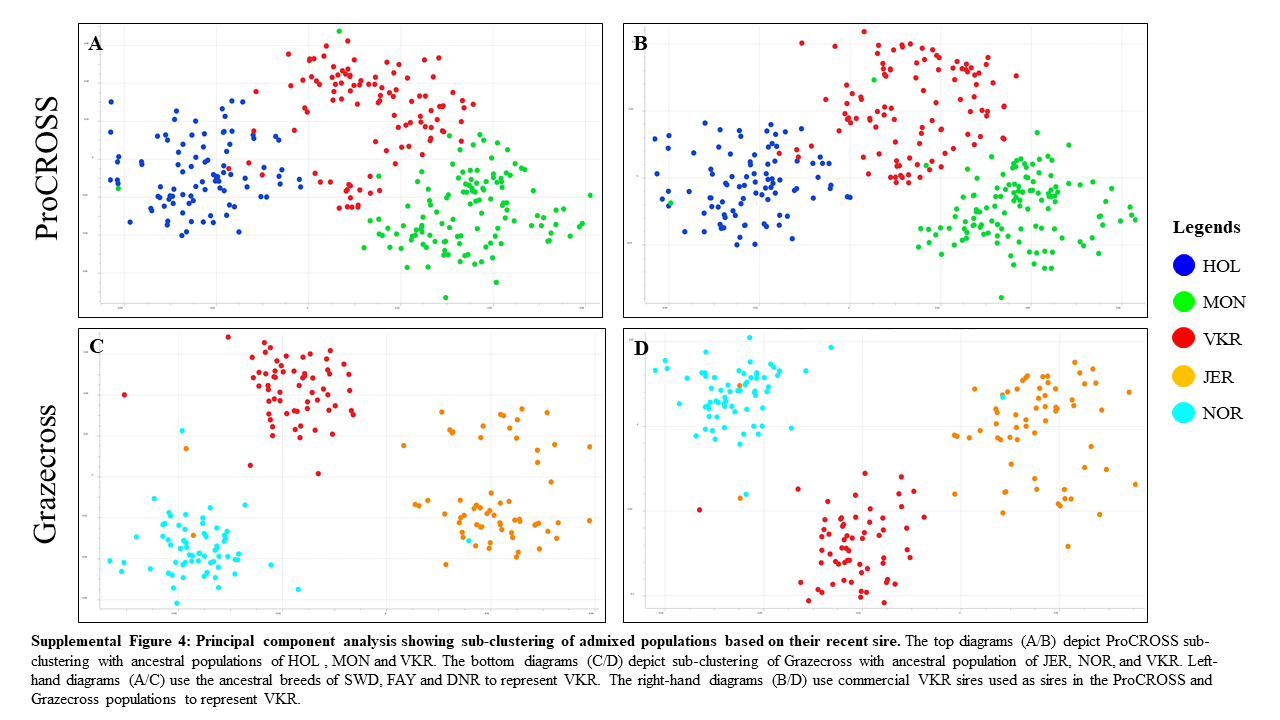

Supplement: Supplementary file 1 [file Image4.PNG]

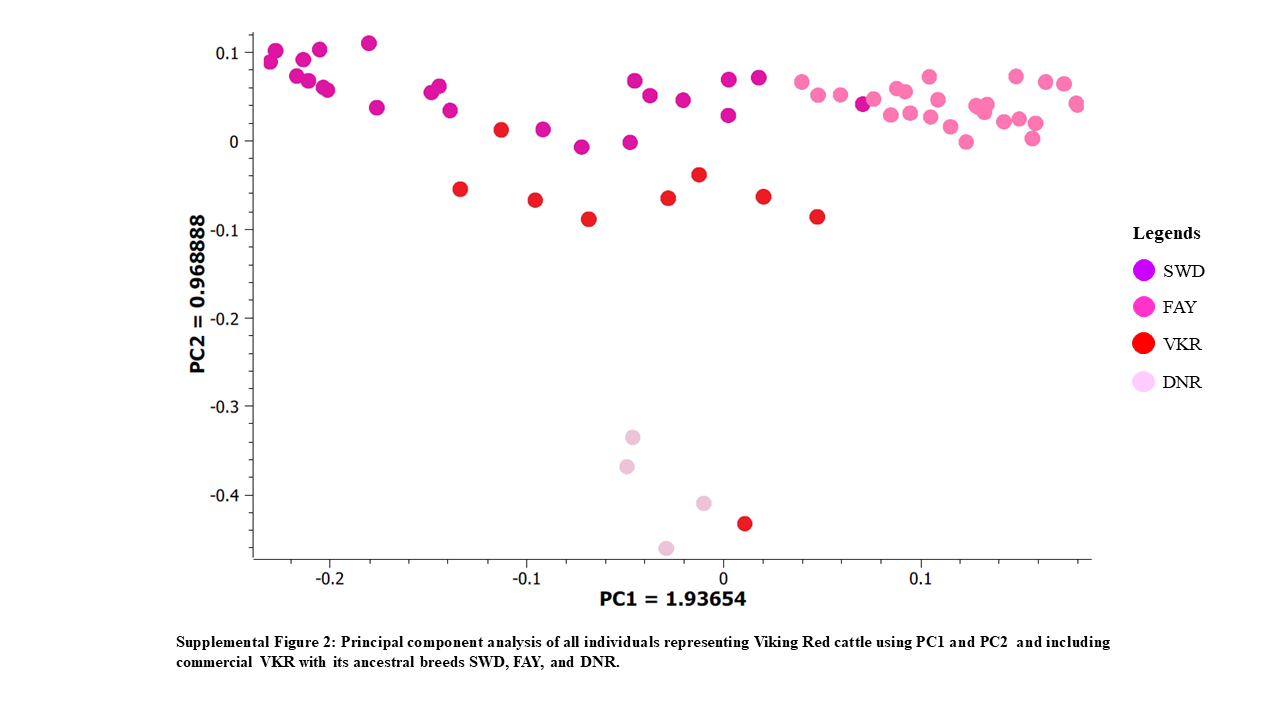

Supplement: Supplementary file 2 [file Image2.PNG]

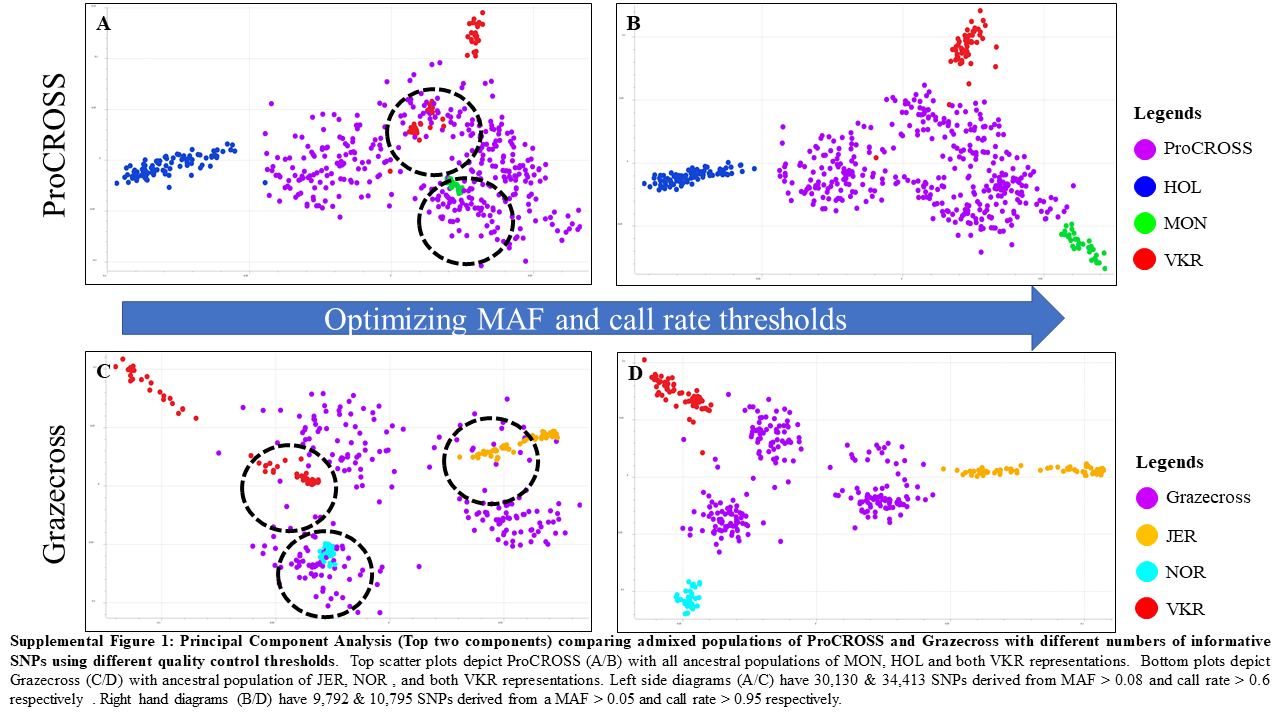

Supplement: Supplementary file 3 [file Image1.PNG]

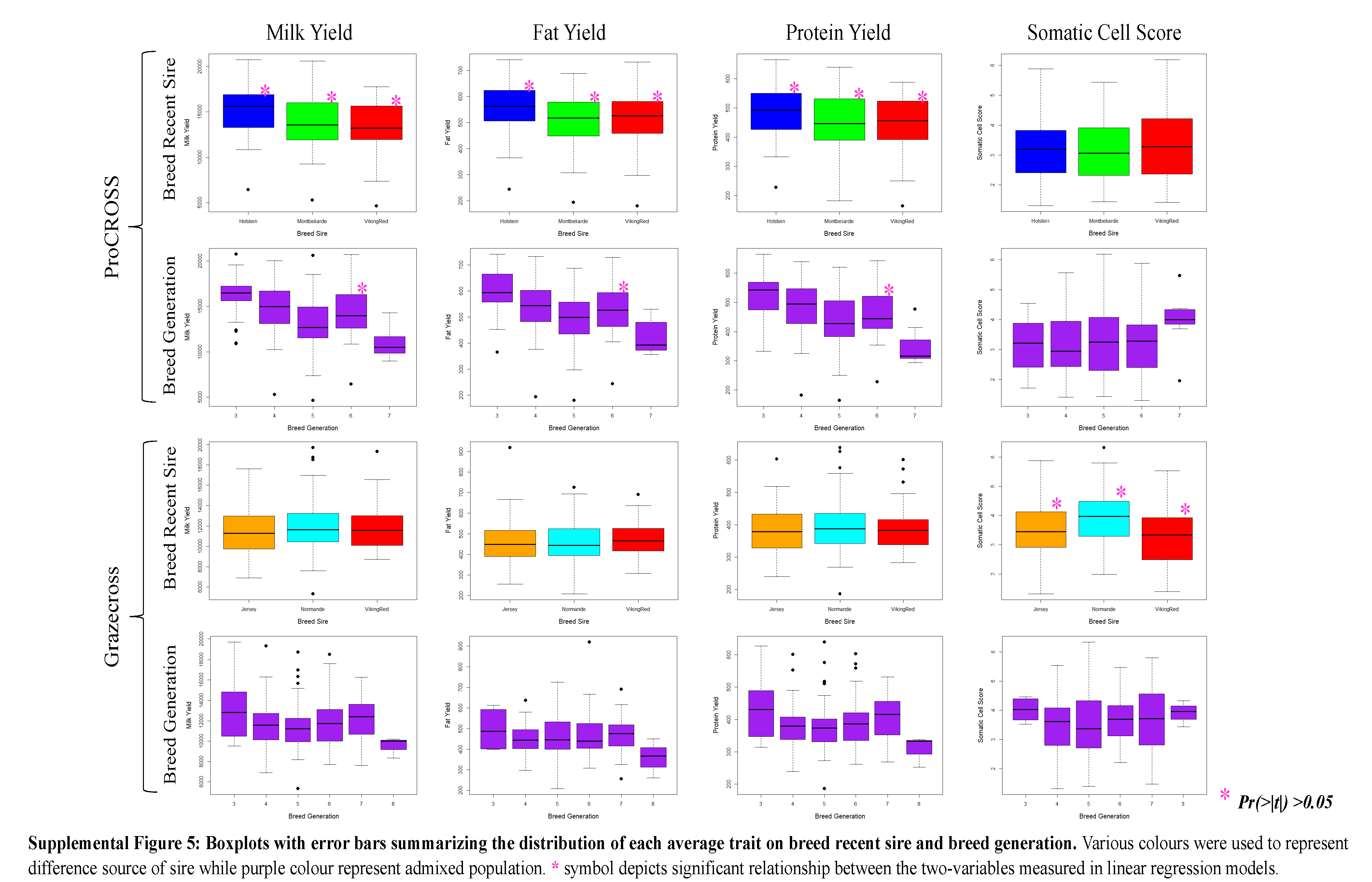

Supplement: Supplementary file 4 [file Image5.TIF]

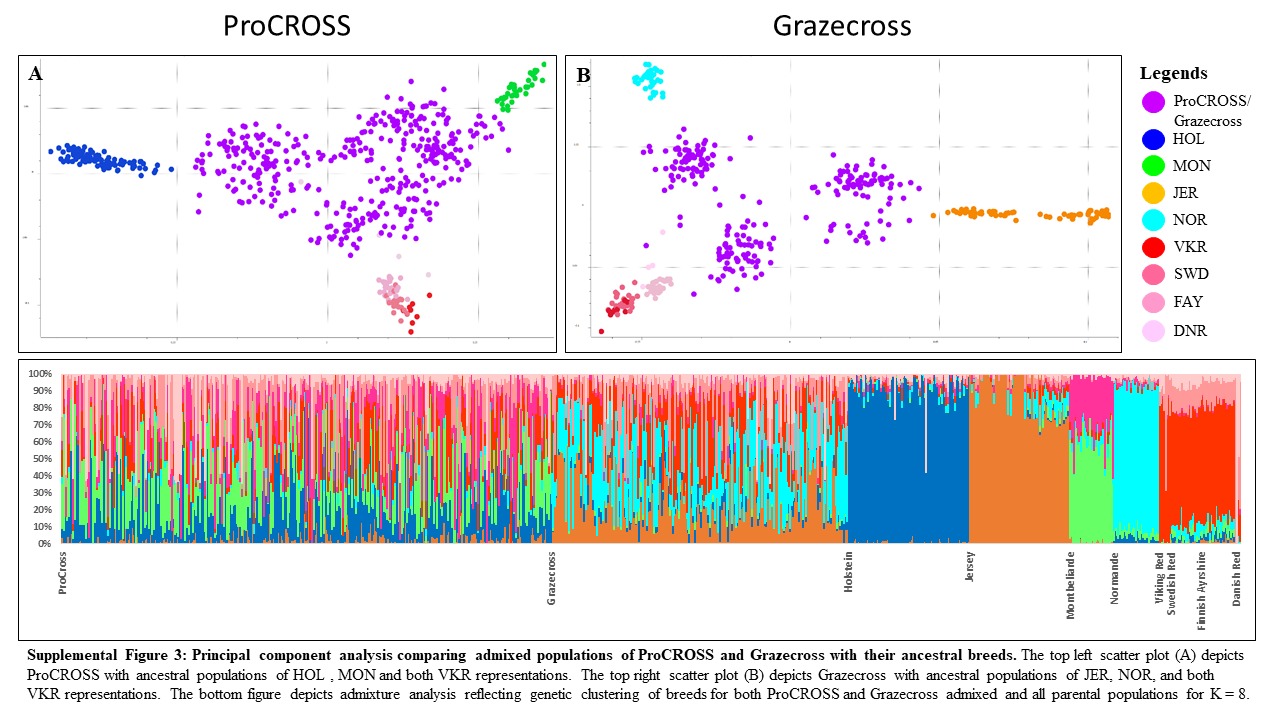

Supplement: Supplementary file 5 [file Image3.PNG]
